# Genetic variant in *CXCL12* gene raises susceptibility to HPV infection and squamous intraepithelial lesions development: a case-control study

**DOI:** 10.1186/s12929-018-0472-y

**Published:** 2018-09-18

**Authors:** Nádia Calvo Martins Okuyama, Fernando Cezar-dos-Santos, Érica Romão Pereira, Kleber Paiva Trugilo, Guilherme Cesar Martelossi Cebinelli, Michelle Mota Sena, Ana Paula Lombardi Pereira, Adriano Martin Felis Aranome, Luis Fernando Lasaro Mangieri, Rodolfo Sanches Ferreira, Maria Angelica Ehara Watanabe, Karen Brajão de Oliveira

**Affiliations:** 1 Laboratory of molecular genetics and immunology, Department of Pathological Science, Londrina State University, Londrina, PR Brazil; 2 Laboratory of study and application of DNA polymorphism, Department of Pathological Science, Londrina State University, Londrina, PR Brazil

**Keywords:** CXCL12, rs1801157 polymorphism, HPV infection, Cervical lesion

## Abstract

**Background:**

Human papillomavirus (HPV) is the most common sexually transmitted virus in women worldwide. The persistence of the virus may cause warts that are considered benign lesions and low or high grade intraepithelial lesions (LSIL/HSIL). Immunological system plays an important role in the resolution of infections. In this context, we highlight the chemokines, which are important regulators in the development of viral infections and inflammation. Among which CXCL12 stands out, due to its pro-inflammatory features, acting as chemoattractant recruiting immune cells. Several polymorphisms were identified in *CXCL12* gene including rs1801157 in the 3′-untranslated region, which is characterized by a substitution of a guanine for an adenine.

**Methods:**

In this study, 195 women were classified as HPV non-infected and 169 as HPV-infected. HPV-DNA was detected by polymerase chain reaction (PCR) and the polymorphism was assessed in blood cells through restriction fragment length polymorphism analysis.

**Results:**

HPV infection was more incident in women who had more than 4 sexual partners during lifetime (*p* = 0.007), among those who presented lower number of pregnancies (*p* = 0.017). HPV was more prevalent among allele A carriers confirmed by logistic regression analysis adjusted for several confounding factors [OR_ADJ_ = 4.985; CI_95%_ (2.85–8.72), *p* < 0.001]. An association between allele A carriers and HSIL development (*p* = 0.003) was also observed.

**Conclusions:**

In the present study, we demonstrated that *CXCL12* rs1801157 is independently associated with HPV infection and exerts influence in HSIL development, suggesting it as a promising susceptibility biomarker for HPV infection and lesions development.

## Background

Human papillomavirus (HPV) is the most common sexually transmitted infection in women worldwide. Infection may resist asymptomatic and is, usually, transient. Most of the women eliminate the virus from the body with the immune system effective action within 5–15 months [1]. The virus persistence may cause warts that are considered benign lesions, low or high grade squamous intraepithelial lesions (LSIL/HSIL) and cancer [2]. Several HPV types, especially high-risk types (HPV-HR), mediate squamous intraepithelial lesion (SIL) development that may progress to cervical cancer through several mechanisms such as keratinocytes malignant transformation, however many other factors contribute to the disease progression, such as tobacco use, long-duration oral contraceptive use and multiparity [3].

Moreover, immunological system plays an important role in the infection resolution. HPV-HR presence may not be elucidated and persist through several years, inducing an inflammatory microenvironment leading to pre-cancerous lesions development [4–6]. It is known that chemokines are important regulators in the development of viral infections [7] and are also responsible for inducing directional keratinocyte migration, notably of leukocytes during inflammation. Prolonged inflammation may facilitate carcinogenesis by providing an ideal microenvironment for tumor growth and development [8]. Several chemokines play important role in inflammation process, including CXCL12 due to its pro-inflammatory characteristic, acting as chemoattractant to immune cells such as lymphocyte [9].

The *CXCL12* gene is located on long arm of chromosome 10 and was first cloned from a bone marrow-derived stromal cell line and then, identified as pre-B cell growth stimulating factor [10]. Several polymorphisms were identified in *CXCL12* gene including rs1801157 in the 3′-untranslated region (3’UTR), described for the first time by Cheryl Winkler in 1998, and is characterized by a substitution from guanine to adenine (g.17289G > A) [11]. This single nucleotide polymorphism (SNP) was associated to elevated risk of some types of cancer development including breast cancer and lymphoma [12]. However, to date, there is no study between *CXCL12* rs1801157 polymorphism and HPV infection as well as cervical lesions development.

In a case-control study the rs1801157 polymorphism was not associated with invasive squamous carcinoma and adenocarcinoma in situ [13]. On the other hand, analysis between this polymorphism genotype distribution and cervical cancer risk, showed that allele A of this polymorphism may be a risk factor for patients with a positive history of tobacco smoking [14].

Due to the lack of data, we aimed to investigate the influence of *CXCL12* rs1801157 polymorphism on HPV infection and LSIL and HSIL development in a Brazilian population.

## Methods

### Ethical approval and sample characterization

This study was approved by Institutional Ethics Committee Involving Humans at State University of Londrina, Londrina – Paraná (PR), Brazil (CEP/UEL 133/2012; CAAE 05505912.0.0000.5231). The study purpose and procedures were explained to all patients and written informed consent was obtained.

Between 2014 and 2016, 364 women were enrolled in this case control-study. They were recruited in health services in Londrina- PR, Brazil: the Intermunicipal Consortium of Health of the Middle Paranapanema, Clinic center of the State University of Londrina, and from two basic health-care units in Londrina – PR, Brazil. After sample collection, cytobrushes containing cervical cells were stored in 2 mL TE buffer (10 mM Tris-HCl, 1 mM EDTA pH 8.0) at − 20 °C until DNA extraction. Peripheral blood was collected with EDTA as anticoagulant and stored at 7 °C. Structured questionnaire was applied concerning sociodemographic, reproductive and sexual behavioral data. Participants were stratified based on HPV DNA presence or absence. Cervical cytology results were collected from medical records.

### Genomic DNA extraction

Genomic DNA was obtained from cervical cytobrushes using DNAzol (Invitrogen™ Inc., Carlsbad, CA, USA) according to the manufacturer’s instructions, and from peripheral blood using Biopur Mini Spin Plus Kit (Biometrix®, Curitiba, PR, Brazil). DNA concentration was measured at 260 nm on a NanoDrop 2000c™ Spectrophotometer (Thermo Fisher Scientific, Walthan, MA, USA), and purity was assessed by absorbance ratio measured at 260 nm and 280 nm.

### HPV detection

**Fig. 1:**
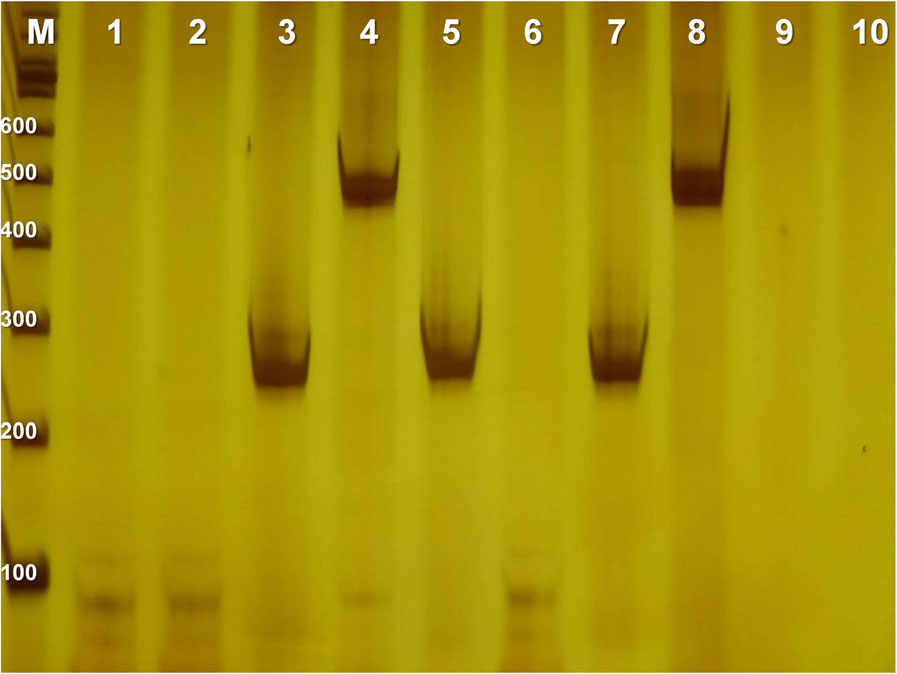
Electrophoretic profile of amplified HPV DNA fragment. 10% polyacrylamide gel stained with silver nitrate. M, 100 bp marker ladder; 1 and 2, negative control for HPV; 3 and 4, positive control; 5 and 6 negative patient for HPV detection; 7 and 8, positive patient for HPV detection; 9 and 10 negative control for β-globin. Each patient has two columns, the first for b-globin (268 bp) and the second for HPV amplification (~450pb)

HPV was detected by Polymerase Chain Reaction (PCR) using the primers MY09 (5’-CGTCCMAARGGAWACTGATC-3′) and MY11 (5’-GCMCAGGGWCATAAYAATGG-3′), which are designed to amplify a conserved region of approximately 450 bp in the HPV L1 gene [15]. Reaction conditions were 190 nM of dNTPs, 500 nM of each primer, 2 mM of MgCl_2_, 1X of Buffer (200 mM Tris-HCL, 500 mM KCl), approximately 80 ng of DNA and 1.25 U of Taq polymerase (Invitrogen™), with an annealing temperature of 55 °C. β-globin gene amplification (268 bp) was performed as an internal control, using primers GH20 (5’-GAAGAGCCAAGGACAGGTAC-3′) and PC04 (5’-CAACTTCATCCACGTTCACC-3′) [16] under the same conditions of HPV PCR. Reactions without template DNA were used as negative control to test for contamination, and DNA from HeLa cells, which are stably integrated with HPV18, was used as positive control. PCR products were electrophoresed on 10% polyacrylamide gel and stained with silver nitrate (Fig. 1).

### *CXCL12* rs1801157 polymorphism genotyping

**Fig. 2:**
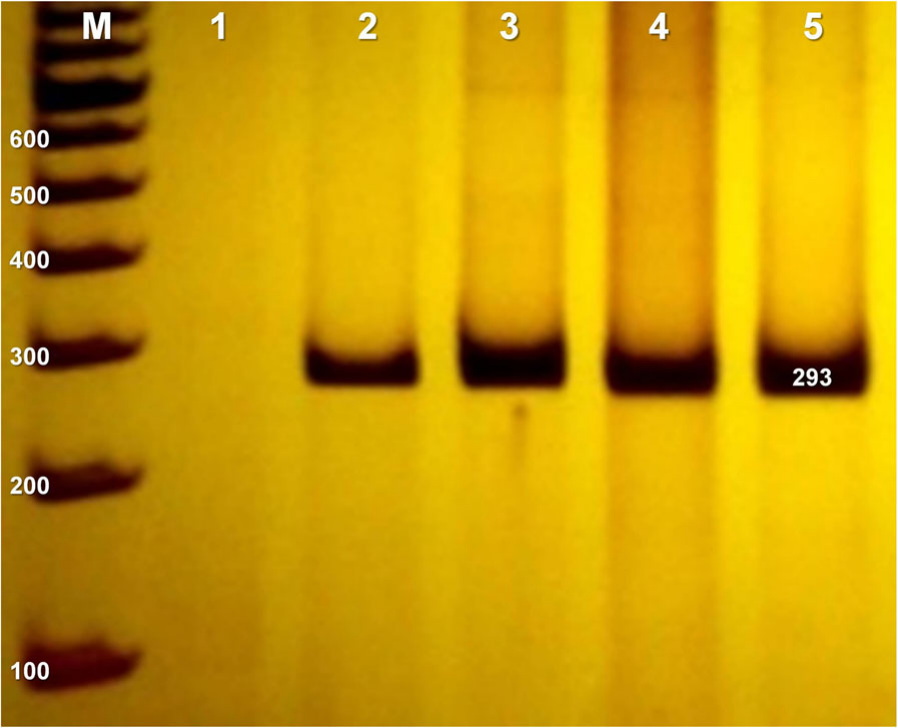
Electrophoretic profile of amplified CXCL12 fragment. 10% polyacrylamide gel stained with silver nitrate. M, 100 bp marker ladder; 1, negative control; Columns 2 to 5, samples showing CXCL12 DNA amplification

Genomic DNA from peripheral blood samples was used to detect *CXCL12* rs1801157 polymorphism by PCR. Primers used for *CXCL12* gene amplification were designed according to the nucleotide sequence deposited in GenBank which code is L36033. The primers forward (5’ CAGTCAACCTGGGCAAAGCC 3′) and reverse (5’ CCTGAGAGTCCTTTTGCGGG 3′) were utilized to amplify part of the 3’UTR of *CXCL12*. PCR conditions were 100 nM of dNTPs, 250 uM of each primer, 1.5 mM of MgCl_2_, 1X of Buffer, approximately 100 ng of DNA and 1 U of Taq polymerase (Invitrogen™) (Fig. 2).

**Fig. 3:**
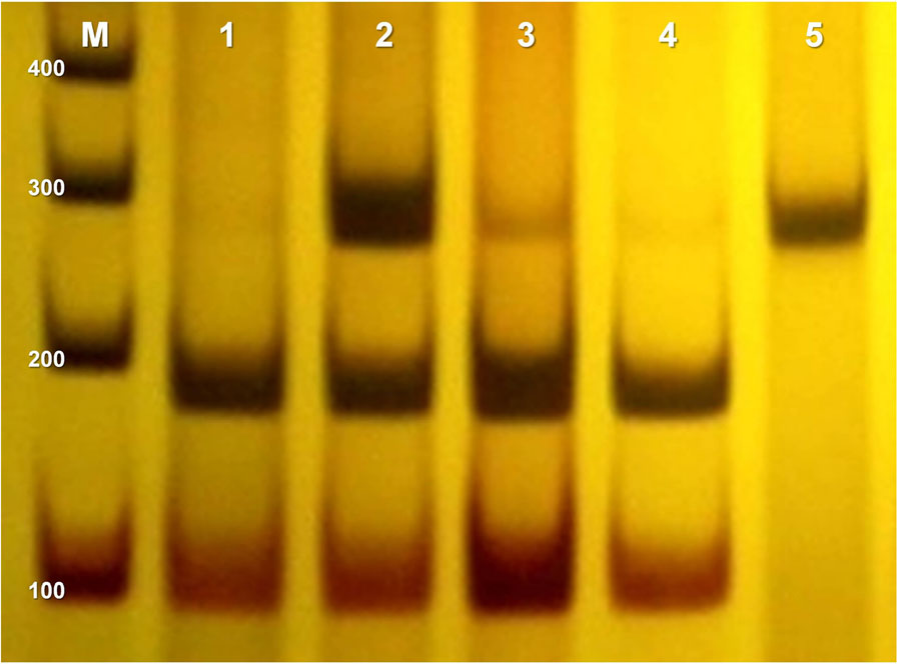
Electrophoretic profile of *CXCL12* rs1801157 polymorphism. 10% polyacrylamide gel stained with silver nitrate. Columns 1, 3, 4: GG Genotype presenting two restriction fragments, 100 bp and 193 bp. Column 2: GA Genotype presenting three fragments, 100 bp, 193 pb and 293 bp. Column 5: AA Genotype showing a single fragment of 293 bp

The *CXCL12* product amplification corresponds to a 293 bp fragment. The enzymatic restriction was performed by PCR-RFLP using PCR product in the presence of the restriction enzyme *MspI* (New England Biolabs, Ipswich, MA, USA). This enzyme cleaves the amplified fragment of DNA in the presence of a guanine, producing fragments of 100 bp and 193 bp and in the presence of an adenine, the fragment of 293 bp remains intact (Fig. 3).

### Statistical analysis

Differences in sociodemographic and sexual behavioral data between infected and non-infected women were examined using contingency tables and Pearson’s x^2^ test. Allele frequency was calculated as [1(h + 2H)]/2 N, where h represents the heterozygous genotype, H is the homozygous genotype, and N is the sample size for each population. Hardy-Weinberg equilibrium in infected and non-infected women was tested using x^2^ test. Differences in the distribution of genotypes were assessed by x^2^ test between non-infected and infected women, and among women with or without low- and high-grade squamous intraepithelial lesions. Adjusted Odds Ratio with 95% confidence interval was calculated to estimate the association between HPV presence, sociodemographic, reproductive and sexual behavior features, as well to analyze association of *CXCL12* polymorphism with HPV presence and lesions development. Binary logistic regression model adjusted for confounding factors was performed to establish the association between HPV presence and *CXCL12* polymorphism. All statistical analysis were performed in SPSS Statistics 22.0 (SPSS Inc., Chicago, Illinois, USA). A *p* value < 0.05 was considered statistically significant.

## Results

In the present study, 364 women were included and categorized as HPV non-infected patients (195/53.6%) and HPV infected patients (169/46.4%) according to the molecular detection of HPV-DNA. Non-infected women mean age was 42 ± 12 years (median = 42), while HPV infected patients mean age was 36 ± 13 years (median = 33).

Sociodemographic characteristics of both groups, HPV infected and HPV non-infected women are presented in Table 1. A higher frequency of HPV was observed in women who had no knowledge about HPV (*p* = 0.024), were younger than 24 years old (*p* = 0.001), single (*p* = 0.002), smokers (*p* < 0.001) and received less than 1 minimum wage (*p* = 0.040).

**Table 1:** Sociodemographic characteristics of HPV positive patients and controls

Variable	HPV non-infected		HPV infected		*p* value*	OR	CI 95%	*p value*
	n	(%)	n	(%)				
Knowledge about HPV					**0.024**			
No	36	(19.35)	43	(32.82)		1.00	Reference	
Have ever heard	106	(57.00)	62	(47.32)		0.490	0.258–0.842	**0.010**
Yes	44	(23.65)	26	(19.86)		0.495	0.257–0.954	**0.036**
Age (years)					**0.001**			
≤ 24	14	(7.21)	30	(18.63)		1.00	Reference	
25–34	50	(25.77)	52	(32.29)		0.483	0.231–1.021	0.057
35–44	50	(25.77)	37	(23.00)		0.345	0.161–0.741	**0.006**
45–54	54	(27.85)	23	(14.28)		0.199	0.088–0.443	**0.001**
≥ 55	26	(13.40)	19	(11.80)		0.341	0.143–0.812	**0.015**
Ethnicity					0.540			
Caucasian	100	(54.06)	58	(44.61)		1.00		
Brown	61	(33.00)	60	(42.60)		1.696	1.048–2.744	**0.031**
Black	24	(13.00)	11	(8.50)		0.790	0.361–1.730	0.556
Asian	0	(0.00)	1	(0.80)		–	–	–
Monthly income^a^					**0.040**			
< 1 minimum wage	43	(24.57)	45	(37.81)		1.00	Reference	
1–3 minimum wages	115	(65.71)	65	(54.62)		0.540	0.322–0.906	**0.020**
3–5 minimum wages	13	(7.42)	9	(7.57)		0.662	0.257–1.706	0.393
≥ 5 minimum wages	4	(2.27)	0	(0.00)		–	–	
Smoking status					**0.001**			
No	163	(84.90)	100	(69.90)		1.00	Reference	
Yes	29	(15.10)	43	(30.10)		2.417	1.419–4.117	**0.001**
Educational Stage^b^					0.488			
Until incomplete fundamental education	58	(31.40)	44	(33.90)		1.00	reference	
Complete fundamental education	17	(9.20)	18	(13.80)		1.396	0.646–3.014	0.396
Incomplete secondary education	29	(15.70)	16	(12.30)		0.727	0.352–1.502	0.390
Complete secondary education	63	(34.10)	42	(32.30)		0.879	0.505–1.528	0.647
Incomplete higher education	6	(3.20)	6	(4.60)		1.318	0.398–4.436	0.651
Complete higher education	12	(6.40)	4	(3.10)		0.439	0.133–1.455	0.178
Marital status					**0.002**			
Single	19	(9.80)	39	(24.80)		1.00	Reference	
Married / Civil partner	143	(73.70)	91	(58.00)		0.310	0.169–0.569	**0.001**
Divorced	23	(11.90)	19	(12.10)		0.402	0.178–0.912	**0.029**
Widowed	9	(4.60)	8	(5.10)		0.433	0.144–1.300	0.136

^a^Based on Brazilian minimum wage (approximately U$ 265.00). ^b^Based on Brazilian educational system. *Analysis by two-sided Chi-square (*Χ*^*2*^) test and *p* < 0.05 set as significance level (SPSS Inc., Chicago, Illinois, USA). Some categories did not complete the total of patients due to lack of data. Significant *p* values are presented in bold

Sexual and reproductive characteristics data are presented in Table 2. HPV infection was more incident in women who had more than 1 sexual partner during lifetime (*p* = 0.007), among those who presented lower number of pregnancy (*p* = 0.017).

**Table 2:** Sexual behavioral and reproductive characteristics of HPV positive patients and controls

Variable	HPV non-infected		HPV infected		*p* value*	OR	CI95%	*p* value
	n	(%)	n	(%)				
Contraceptive method					0.216			
No	114	(59.70)	83	(54.20)		1.00	Reference	
Yes, hormonal	63	(33.00)	52	(34.00)		1.134	0.713–1.802	0.569
Yes, condom	13	(6.80)	13	(8.50)		1373	0.605–3.116	0.448
Yes, both	1	(0.50)	5	(3.30)		6.867	0.788–59.882	0.081
Number of pregnancies					**0.017**			
0	15	(7.70)	26	(16.30)		1.00	Reference	
1	33	(17.00)	42	(26.30)		0.451	0.243–0.835	**0.011**
2	61	(31.50)	35	(21.90)		0.540	0.286–1.020	0.058
3	48	(24.50)	33	(20.60)		0.499	0.193–1.043	0.063
4	21	(10.80)	12	(7.50)		0.589	0.245–1.146	0.237
≥ 5	16	(8.50)	12	(7.40)		1.362	0.623–2.977	0.439
Abortion					0.092			
No	137	(78.28)	107	(80.45)		1.00	Reference	
Yes	38	(21.72)	26	(19.55)		0.876	0.501–1.533	0.643
Age at first sexual intercourse (years)					0.265			
≤17	102	(53.70)	89	(59.70)		1.00	Reference	
≥18	88	(46.30)	60	(40.30)		0.782	0.506–1.206	0.266
Age at menarche (years)					0.379			
≤12	89	(53,65)	82	(54,67)		1.00	Reference	
≥13	103	(46,35)	68	(45,33)		0.717	0.467–1.101	0.128
Sexual partners during the lifetime					**0.007**			
1	76	(40.60)	31	(23.80)		1.00	Reference	
2–3	53	(28.40)	44	(33.80)		2.035	1.142–3.628	**0.016**
≥4	58	(31.00)	55	(42.40)		2.325	1.332–4.059	**0.003**
Sexual partners within the past 6 months					0.529			
0	26	(13.90)	24	(18.20)		1.00	Reference	
1	158	(84.50)	103	(78.00)		0.706	0.385–1.297	0.262
≥2	3	(1.60)	5	(3.80)		1.806	0.389–8.382	0.451

*Analysis by two-sided Chi-square (*Χ*^*2*^) test and *p* < 0.05 as significance level (SPSS Inc., Chicago, Illinois, USA). Some categories did not complete the total of patients due to lack of data. Significant *p* values are presented in bold

*CXCL12* rs1801157 polymorphism genotypes distribution among HPV non-infected and infected patients were in Hardy-Weinberg equilibrium (*p* ≥ 0.05). A higher frequency of allele A was observed in HPV infected women (p < 0.001) which was confirmed by codominant, dominant and recessive models (Table 3). Considering the allele A variant incidence in both populations, and the number of enrolled women, the power of our analysis was calculated as 99.95%.

**Table 3:** Association between *CXCL12* rs1801157 polymorphism and HPV infection

Model	HPV non-infected N (%)	HPV infected N (%)	OR	CI95%	*p* value
Codominant model					
GG	147 (75.40)	71 (42.00)	1.00		
GA	45 (23.10)	71 (42.00)	**3.25**	**2.044–5.22**	**< 0.001**
AA	3 (1.50)	27 (16.00)	**18.63**	**5.47–63.49**	**< 0.001**
Dominant model					
GG	147 (75.40)	71 (42.00)	1.00		
GA + AA	48 (24.60)	98 (58.00)	**4.22**	**2.70–6.60**	**< 0.001**
Recessive model					
GG + GA	192 (98.50)	142 (84.00)	1.00		
AA	3 (1.50)	27 (16.00)	**11.80**	**3.55–39.56**	**< 0.001**
Alleles					
G	339 (86.92)	213 (63.02)	1.00		
A	51 (13.08)	125 (36.98)	**3.90**	**2.70–5.63**	**< 0.001**

Analysis by two-sided Chi-square (Χ^*2*^) test (*p* < 0.05 as significance level). *OR* Odds Ratio; *CI* Confidence Interval. (SPSS Inc., Chicago, Illinois, USA). Significant *p* values are presented in bold

In order to confirm whether *CXCL12* rs1801157 polymorphism is associated with infection independently of confounding factors, data were adjusted for all confounding factors observed in the previous analysis in a binary logistic regression (Table 4). A significant association between allele A and HPV infection was confirmed in all the seven models proposed, indicating that the polymorphism is independently associated to HPV infection. As observed in model 7, in which data was adjusted for knowledge about HPV, age, monthly income, smoking status, number pregnancies, number of sexual partners, and marital status allele A carriers presented an increased risk for HPV infection [OR_ADJ_ = 4.947; CI_95%_ (2.854–8.575), *p* < 0.001].

**Table 4:** Association study between CXCL12 rs1801157 and HPV in dominant model infection adjusted for confounder factors

	Model 1	Model 2	Model 3	Model 4	Model 5	Model 6	Model 7
GG							
OR	1.00	1.00	1.00	1.00	1.00	1.00	1.00
CI95%	Reference	Reference	Reference	Reference	Reference	Reference	Reference
*p* value							
Allele A carrier							
OR	**4.004**	**4.416**	**4.878**	**4.836**	**4.824**	**4.887**	**4.947**
CI95%	(2.472–6.483)	(2.677–7.284)	(2.865–8.308)	(2.819–8.296)	(2.811–8.279)	(2.829–8.442)	(2.854–8.575)
*p* value	**< 0.001**	**< 0.001**	**< 0.001**	**< 0.001**	**< 0.001**	**< 0.001**	**< 0.001**

Logistic regression analysis with HPV as dependent variable (reference group = non-infected women) and CXCL12 rs1801157 polymorphism as explanatory variable, adjusted for several con- founders according to the proposed models (*p* < 0.05 as significance level). (SPSS Inc., Chicago, Illinois, USA). Significant *p* values are presented in bold

Model 1: CXCL12 polymorphism adjusted for knowledge about HPV;

Model 2: CXCL12 polymorphism adjusted for knowledge about HPV and age;

Model 3: CXCL12 polymorphism adjusted for knowledge about HPV, age and monthly income;

Model 4: CXCL12 polymorphism adjusted for knowledge about HPV, age, monthly income and smoking status;

Model 5: CXCL12 polymorphism adjusted for knowledge about HPV, age, monthly income, smoking status and number of pregnancies;

Model 6: CXCL12 polymorphism adjusted for knowledge about HPV, age, monthly income, smoking status, number of pregnancies, and number of sexual partners;

Model 7: CXCL12 polymorphism adjusted for knowledge about HPV, age, monthly income, smoking status, number of pregnancies, number of sexual partners and marital status

Considering the polymorphism influence in lesions development, the dominant model was adopted in order to make a better distribution among genotype groups (Table 5). We observed that allele A presence was not associated to LSIL (*p* = 0.476) compared to women without lesion. However, it was significantly associated to HSIL (*p* = 0.003) development.

**Table 5:** Association study between CXCL12 rs1801157 allele A and lesion development

	n (%)	OR	CI95%	*p* value
LSIL				
GG	15 (57.70%)	1.000	Reference	
GA + AA	11 (42.30%)	1.347	0.594–3.054	** 0.476**
HSIL				
GG	32 (45.10%)	1.000	Reference	
GA + AA	39 (54.90%)	**2.239**	**1.315–3.811**	**0.003**

Data were analyzed compared to patient without lesion. *LSIL* Low-grade squamous intraepithelial lesion. *HSIL* High-grade squamous intraepithelial lesion. *OR* Odds Ratio. *CI* Confidence Interval. (SPSS Inc., Chicago, Illinois, USA). Significant *p* values are presented in bold

## Discussion

To the best of our knowledge, this is the first study that demonstrated an independent association between *CXCL12* rs1801157 polymorphism HPV infection and HSIL.

According to sociodemographic data, HPV was more frequent within patients who had no knowledge about the virus which may indicated lack of information about HPV and also, the ways to avoid virus exposure; among women younger than 24 years old, single and who had more than 1 sexual partner during lifetime.

Young age has been associated in an independent way to HPV infection [2] and it is also in accordance to Sanjosé meta-analysis [17], probably due to the intense sexual activity among younger women, besides that, younger women usually present a larger area of ectopy compared with older adults, what means biological vulnerability to HPV infection because of the easier access to basal epithelial cells [18].

In this study, smoking status was associated to HPV infection (*p* < 0.001), this could be explained by the fact that tobacco smoking may cause immunosuppression [19, 20]. Smoking may inhibit the immune response to HPV by decreasing Langerhans’ cell in normal epithelium, moreover HPV-infected cells are exposed to tobacco carcinogens that cause DNA damage while HPV oncoprotein E6 block apoptosis [19]. Alam et al. [21] also reported a molecular interaction between benzo[a]pyrene (BaP), a carcinogen found in tobacco smoke, and HPV synthesis, suggesting that BaP might interfere on multiple HPV life cycle functions, such as inducing genome copies, stimulating and/or stabilizing late gene transcripts/capsid proteins and the concomitant virion assembly, potentially enhancing viral persistence, host tissue carcinogenesis, and permissiveness for cancer progression.

Lower pregnancies number (*p* = 0.017) was also associated to HPV presence. During pregnancy, elevated estrogen and progesterone may lead to the squamous-columnar junction exposure and metaplasia. Parity might increase the risk of cervical cancer because it maintains the cervix transformation zone for many years, facilitating exposure to HPV infection and others cofactors [22]. Another factor that contributes to HPV infection in pregnant women is the immunosuppression due to the steroid hormones increased levels that depress cellular immunity [23] and may also have an effect on HPV replication. Besides, it has been shown that the transcriptional promoter of E6-E7 transforming region of HPV16 contains a steroid hormone receptor-binding element that stimulates HPV E6 and E7 transcription, suggesting a hormonal activation effect on HPV replication [24]. Nonetheless, our data have demonstrated high risk of infection in women with no pregnancies. This might be explained by the fact that the young age of our patients is correlated to lower number of pregnancies (data not shown) as well as higher risk of infection.

Genetic factors have been suggested to play a role in HPV persistence besides environmental and lifestyle factors [25]. Virus persistence and cervical cancer risk may vary among individuals and can be partly explained by individual variations in genes involved in this complex mechanism. A combination of several genetic variants may modulate the risk factors, therefore the identification of susceptibility alleles remains a promising research field [26].

In this context, we analyzed the *CXCL12* rs1801157 polymorphism in HPV infection, LSIL and HSIL development. A higher frequency of HPV was observed among allele A carriers, confirmed by binary logistic regression model adjusted for several factors as confounders, demonstrating that *CXCL12* rs1801157 is independently associated to HPV infection. Some studies observed that the polymorphism was not a risk factor for cervical cancer development [13, 14, 27] however, none of them have evaluated whether the polymorphism could represent a risk factor for HPV infection as demonstrated in this study.

Precursor lesions can occur as consequence of persistent infection. In the present study, allele A influence in cervical lesion development was also evaluated and a significant association was observed for allele A carriers with HSIL (*p* = 0.003). Increased gradient of CXCL12 concentration was observed from LSIL to HSIL in women with HR-HPV [28]. Hence, would be expected that allele A carriers were also significantly more frequent in LSIL group, but we did not verify this association. It occurs probably due to the reduced number of our LSIL sampling, which may lead to a lesser analytical power. Further investigation with a larger SIL sampling is currently being performed to confirm this assumption.

CXCL12 has been considered as a standard proinflammatory molecule for a long time, since it attracts leukocytes to inflammatory sites contributing to their activation [9, 29]. Data have suggested that HPV pre-cancerous lesion depend on both the suppression of cellular immunity, driven by the Th1 response and the development of the immunosuppressive Treg profile for neoplastic progression [30]. Significant increased expression of CXCL12, measured by IHC and ELISA, in cervical epithelium, as the neoplastic lesion progressed from preinvasive to invasive cancer, was shown by Jaafar et al. [31]. They also showed that CXCL12 was not expressed in normal cervical squamous or glandular epithelium, which is in accordance with Zanotta et al. [32] who have shown that healthy cervical tissue presented low or no levels of CXCL12. A particular significance correlation was found between CXCL12 and FOXP3 in cervical neoplastic lesion, suggesting that high levels of CXCL12 leads to retention or accumulation of FOXP3^+^ T cells in progressing cervical cancer [31].

Until present there are no studies about the *CXCL12* rs1801157 polymorphism influence in its expression, plasmatic or cervical levels in HPV infection or SIL development, but it has been widely studied in others diseases and tumors, showing conflicting results. De Oliveira et al. [12] demonstrated that allele A carriers breast cancer patients have significant low levels of *CXCL12* mRNA in the peripheral blood samples when compared to GG patients. Controversially, Hirata et al. [32] observed in prostate cancer patients that CXCL12 expression was higher in A allele than in allele G carriers.

Immunohistochemistry profile of CXCL12 in colorectal cancer showed weak or negative in normal mucosa and strongly increased in cancer tissues especially in well-differentiated tumors, 73.5% of patients that expressed a strong CXCL12 immunostaining in the membrane and cytomembrane presented AA or GA genotype. By the other hand, 88.6% of those with negative immunoreactivity presented GG genotype [33]. However, in another study with colorectal cancer patients, CXCL12 plasma levels were not related to A allele or GA / AA genotypes [34].

The 3′ untranslated region of genes contains several regulatory motifs that is target of posttranscriptional regulation through interaction with microRNAs, RNA-binding proteins, and long non-coding RNAs, which influence on mRNA turnover, stability and localization. SNPs located in these motifs may prevent miRNA binding and cause mRNA transcript stabilization and increased protein expression [35]. Although the controversial nature of reports about *CXCL12* rs1801157 SNP impact on chemokine levels [36–39], evidence indicate that miRNAs are involved in protein production and regulation. In silico analysis revealed a seven bases long homologous sequence where rs1801157 is located (i.e., the 4th base is the polymorphic one), which is a putative target of miR941. Thus, the SNP presence may cause loss of miRNA941 binding site. However, interaction analysis between miR941 and the 3’UTR in stem cells from healthy donors was assessed by dual luciferase assays and 3’UTR expression was not affected by co-expression of miR941 [40]. Available knowledge about 3’UTR and miRNAs interaction is poor and deserves better understanding.

Evidence for the involvement of the CXCL12 in the HPV life cycle arose from the abnormal and specific expression of CXCL12 observed in keratinocytes of HPV-productive skin or mucosal lesions [41].

CXCL12 expression levels may increase in keratinocytes as a consequence of HPV genome expression, generating an autocrine signaling loop essential for keratinocyte proliferation and migration [42]. A reasonable explanation for this mechanism is that the *CXCL12* proximal promoter in its 5′-flanking and 5′-untranslated region contain six Sp1 binding sites, and Sp1 transcription factor seems to be the major positive regulator of *CXCL12* expression [43]. Additionally, after HPV infection of basal epithelial cervical cells, E6 and E7 oncoproteins are expressed, and may bind specifically to protein 1 transcription factor (Sp1). The E6-Sp1 and E7-Sp1 complex can migrate into the nucleus and probably induce the *CXCL12* gene expression [44].

## Conclusions

More studies are necessary to determine the rs1801157 polymorphism influence in CXCL12 expression and in its cervical levels, in order to establish its influence in HPV infection and in cervical lesion development. However, our work is pioneer in demonstrating the association of *CXCL12* rs1801157 polymorphism to HPV infection and HSIL, suggesting it as a promising susceptibility biomarker for HPV infection and the development of cervical lesions.

## Abbreviations

3’UTR: Untranslated Region
CEP: Comitê de Ética em Pesquisa – Universidade Estadual de Londrina
CXCL12: Chemokine ligand (family CXC) 12
DNA: Desoxyribonucleic acid
dNTP: Deoxynucleotide triphosphate
E: Early
EDTA: Ethylenediaminetetraacetic acid
HPV: Human Papillomavirus
HPV-HR: HPV High Risk
HR: High risk
HSIL: High-grade intraepithelial lesion
KCI: Potassium chloride
L: Late
LCR: Long control region
LSIL: Low-grade squamous intraepithelial lesion
mRNA: Messenger RNA
PCR: Polymerase chain reaction
PCR-RFLP: Restriction fragment length polymorphism
SDF-1: Stromal cell-derived factor 1
SNP: Single nucleotide polymorphism
Sp1: Specificity protein 1 transcription factor
TE: Tris-HCL-EDTA
